# Providing resource to hire “extra hands” as a strategy to retain funded research faculty during periods of significant caregiving responsibilities.

**DOI:** 10.1017/cts.2025.10126

**Published:** 2025-08-26

**Authors:** Madeline Gibson, Naomi Duffort, Reid Eagleson, Richard Kennedy, Rebecca Reamey, Katerine Meese, Bertha Hidalgo, Alia Tunagur, Michael Mugavero

**Affiliations:** 1 Center for Outcomes and Effectiveness Research and Education, University of Alabama at Birmingham, Birmingham, AL, USA; 2 Department of Medicine, University of Alabama at Birmingham, Birmingham, AL, USA; 3 Department of Family and Community Medicine, University of Alabama at Birmingham, Birmingham, AL, USA; 4 Department of Health Services Administration, University of Alabama at Birmingham, Birmingham, AL, USA; 5 Department of Epidemiology, University of Alabama at Birmingham, Birmingham, AL, USA

**Keywords:** Research development, caregiving, physician scientist, early-career investigator, retention, pre-retention

## Abstract

Tension between professional obligations and extraprofessional caregiving responsibilities is one reason physician scientists leave academic medicine. The COVID-19 pandemic exacerbated this challenge by increasing caregiving demands and decreasing time spent on research as much as 40%. *CARES at UAB* (Caregiving Affected Research Early-Career Scientists Retention Program at the University of Alabama at Birmingham) provided “extra hands” awards to early-career physician and non-physician research faculty to hire personnel to expedite research projects already awarded but deleteriously affected by caregiving during the pandemic. Evaluation included tracking awardee publications and grants, surveying awardees, and conducting semi-structured individual in-depth interviews. *CARES at UAB* distributed 28 grants totaling $1,005,266. Twenty-six awardees (93% retention) remain in academia 2.25–3.25 years after award initiation. Awardees attribute over 200 manuscripts to the funding and have secured 15 new NIH K-, R-, and U-series grants. Surveys indicate improved awardee well-being and decreased caregiving burden since receipt of funding. Scientific productivity, feeling valued, sense of community, and lifeline emerged as themes from interviews. Group “listening sessions” yielded university-level recommendations around tenure and promotion, caregiving culture, and mentoring. Resource to hire “extra hands” holds promise to retain early-career physician and non-physician research faculty with extraprofessional caregiving responsibilities.

## Introduction

Over 40% of physicians leave academia within 10 years of their first appointment as an assistant professor at an academic medical center (AMC). Attrition rates are even higher for women and non-white persons (45% each) [1]. Although a myriad of circumstances contributes to this phenomenon, difficulty navigating the integration of extraprofessional caregiving responsibilities and workplace expectations is one of them [2]. Extraprofessional caregiving responsibilities (henceforth: “caregiving responsibilities”) often include childbirth, routine childcare and/or eldercare, and care for spouses and/or other family members in declining health. For research faculty with caregiving responsibilities, the COVID-19 pandemic amplified and illuminated the tension between caregiving obligations and sustained scientific productivity [3]. Many academic institutions and federal agencies responded with extended deadlines, lowered productivity expectations, and extended timelines for promotion and tenure. However, such accommodations did not address the root challenges faced by research faculty with caregiving responsibilities: namely, a decrease in time spent on research by as much as 40% [4].

In 2015, Doris Duke Foundation established the *Fund to Retain Clinical Scientists* (*FRCS*) to sponsor institutional-level “extra hands” programs to help early-career physician scientists sustain research productivity when faced with caregiving responsibilities [5,6]. The FRCS also increased national awareness about funding support for personnel to perform research activities as one method of reducing strain and retaining early-career physician scientists with caregiving responsibilities in academic medicine. In 2021, the Foundation partnered with the American Heart Association, the Burroughs Wellcome Fund, Rita Allen Foundation, and Walder Foundation to expand the program in light of the enormous tension between research productivity and family caregiving during the COVID-19 pandemic [7]. The *COVID-19 Fund to Retain Clinical Scientists (COVID-19 FRCS)* solicited applications from U.S. medical schools and provided grants of $550,000 over two years. Twenty-two medical schools received *COVID-19 FRCS* funding. In this Special Communications, we describe the *COVID-19 FRCS* program at the University of Alabama at Birmingham (UAB) and its preliminary results.

To our knowledge, this evaluation is the first on awardees supported through *COVID-19 FRCS* and first to report on any *FRCS* program at an institution in the historical U.S. Deep South. Unlike other *FRCS* programs, *CARES at UAB* utilized institutional support to expand awardee eligibility and subsequent evaluation to non-physician research faculty who otherwise met eligibility criteria in both Schools of Medicine and other health sciences. *CARES at UAB* also limited allowable expenses to delegable research tasks and did not allow faculty to “buy out” their own time from clinical or other university obligations. In so much, this Special Communications builds upon previous *FRCS* program evaluations and adds new insights to the available literature.

## Materials and methods

At UAB, we branded the *COVID-19 FRCS* grant as Caregiving Affected Research Early-career Scientists Retention Program (henceforth: *CARES at UAB*). The initial call for applications was limited to criteria set forth by the Doris Duke Foundation: funded (50% effort or greater) early-career physician scientists whose research productivity was deleteriously affected by caregiving responsibilities during the height of the COVID-19 pandemic. However, as a result of the robust internal interest from early-career non-physician scientists who also faced challenges with work-life integration during this period and through the provision of intramural funds by the UAB Heersink School of Medicine (HSOM), UAB School of Public Health, UAB School of Nursing, UAB School of Health Professions, UAB Department of Pediatrics, and UAB Center for Clinical and Translational Science (totaling $650,000), we made a second and third call for applications from both physician scientists and their non-clinical counterparts in the four participating schools. Applications were scored by UAB faculty according to impact of caregiving responsibilities, immediacy of external funding termination, and record of pre-pandemic productivity. Awards began January 1, 2022 (HSOM physician scientists); October 15, 2022 (HSOM physician scientists and non-physician scientists in Public Health, Health Professions, and Nursing); and January 1, 2023 (HSOM non-physician scientists). Awards provided up to $50,000 in direct costs for an 18-month award period, including a 6-month no-cost extension. Allowable costs included effort for qualitative and quantitative methodologists, graduate students, study coordinators, and scientific editors and writers, to name a few. Awardee effort, travel, and other expenses that did not directly help applicants reclaim their research progress were not allowed.

In addition to the discretionary funding to hire “extra hands,” we required awardees to attend a monthly, virtual, one-hour career development seminar as a cohort. Seminars were split between career development topics (e.g., how to build a research team) and awardee works-in-progress presentations. Organically, Round 2 and 3 awardees expressed a desire to create a “safe space” where they could discuss the challenges they face as early-career research faculty with caregiving responsibilities and ways the university could address these challenges. We devoted two half-hour sessions to this topic during the seminar series. We collated the challenges and recommendations shared during these “listening sessions” and asked awardees to approve the de-identified summary before sharing it with university leadership. The insights were used solely as recommendations to university leadership and were not analyzed in conjunction with other evaluation data.

We evaluated the initial impact of the program through several methods. We utilized the Doris Duke Foundation’s progress report template to annually collect manuscripts resulting from *CARES at UAB* funding. We searched NIH Reporter for awardees’ receipt of subsequent NIH awards. To assess well-being, perceptions of institutional support, and caregiver burden, we surveyed awardees at the time of application (December 2021, August 2022, and November 2022) and in July–October 2024 using the Physician Well-Being Index (PWBI) [8], 8 items from the Perceived Organizational Support (POS) scale [9], and the modified Burden Scale for Family Caregivers-Short form (BSFC-s) [10]. All questionnaires used Likert scales, with scores ranging from 0 to 7 for the 7-item PWBI (higher scores indicating poorer quality of life), from −2 to 9 for the 9-item PWBI, from 10 to 50 for POS (higher scores indicating greater organizational support), and from 10 to 50 for the modified BFSC-s (higher scores indicating greater burden). We calculated summary statistics for the entire sample at baseline using means and standard deviations for continuous variables and frequencies and percentages for categorical variables. We assessed changes in questionnaire responses from baseline to follow-up using paired *t*-tests and Wilcoxon’s tests. We did not survey unfunded applicants at follow-up as we did not want to place additional burden on faculty who had already expressed challenges navigating work-life integration.

To understand program impact, we conducted semi-structured interviews between August 2023 and April 2024, 7–28 months after award initiation, with awardees remaining at UAB (*n* = 23). The *CARES at UAB* non-clinical program manager (N.D.) with a Master of Social Work conducted all of the interviews on Zoom. All interviewees provided verbal consent at the beginning of the Zoom session.

We coded and analyzed the interviews using thematic analysis. Two analysts, the *CARES at UAB* program manager and an additional non-clinical research person with expertise in qualitative analysis (R.E.), independently coded the transcripts in NVivo^TM^. The codebook was built from an a priori set of codes developed from the interview guide, initial impressions from the interviews, and existing literature. After coding the first two transcripts independently, coders met to create additional codes to provide greater specificity in identifying themes. They also met iteratively throughout the coding process to discuss codes and themes and resolve differences.

The UAB Institutional Review Board approved our conduct of the survey and semi-structured interviews (IRB-300010606). The conduct of the two group listening sessions was determined to be quality improvement, outside the purview of the UAB IRB, according to UAB’s Quality Improvement Self-Determination Tool.

## Results

*CARES at UAB* received 32 applications and funded 28 “extra hands” awards, as UAB provided more than 1:1 institutional funds to match *COVID-19 FRCS* funds. Awardees were 71% women and 21% persons from backgrounds traditionally underrepresented in biomedical sciences. Fourteen (50%) were physician scientists, and fourteen (50%) held non-clinical doctoral degrees (see Table 1 for additional awardee characteristics). Awards ranged from $10,000–$50,000 and totaled $1,005,266. As of April 4, 2025 (2.25–3.25 years follow-up), twenty–six (93%) awardees remain in academia, with 23 (82%) remaining at UAB. One physician and one non-physician left academia. Awardees attribute over 200 manuscripts to the funding. Awardees also secured 5 new K-series awards and 10 new R- and U-series awards as of April 4, 2025. All remaining at UAB were interviewed (*n* = 23); one did not complete the follow-up survey (*n* = 22).

**Table 1.** Participant characteristics

Baseline	*n* (%)	*N*
Terminal degree(s):		28
MBBCH, PhD	1 (3.57%)	
MBBS	1 (3.57%)	
MD	8 (28.6%)	
MD, PhD	4 (14.3%)	
PhD	14 (50%)	
Gender:		28
Women	20 (71.4%)	
Men	8 (23.8%)	
Race/Ethnicity:		28
Asian	3 (10.7%)	
Black or African American	5 (17.9%)	
Hispanic or Latino	1 (3.57%)	
Indian	1 (3.57%)	
Middle Eastern	1 (3.57%)	
More than one race	1 (3.57%)	
White	16 (57.1%)	
School:		28
School of Health Professions	5 (17.9%)	
School of Medicine	18 (64.3%)	
School of Nursing	3 (10.7%)	
School of Public Health	2 (7.14%)	
Rank:		28
Assistant Professor	19 (67.9%)	
Associate Professor	9 (32.1%)	
Physician Well-Being Index, 7 Item	4.36 (1.39)	28
Physician Well-Being Index, 9 Item	3.61 (1.87)	28
Perceived Organizational Support	24.9 (1.59)	27
Modified Burden Scale for Family Caregivers-Short	36.8 (7.08)	28

Change over time					
	Baseline		Follow-Up		
	Mean (SD)	*N*	Mean (SD)	*N*	*P* Value
Physician Well-Being Index, 7 Item	4.27 (1.49)	22	3.05 (1.70)	22	0.02
Physician Well-Being Index, 9 Item	3.45 (1.97)	22	1.82 (2.17)	22	0.01
Perceived Organizational Support	24.9 (1.70)	21	24.0 (2.00)	21	0.12
Modified Burden Scale for Family Caregivers- Short	37.0 (7.52)	22	30.1 (8.76)	20	0.01

At the time of initial application, awardees reported a mean (SD) 7-item PWBI of 4.36 (1.39), a 9-item PWBI of 3.61 (1.87), POS of 24.9 (1.59), and modified BSFC-s of 36.8 (7.08). Awardees completed follow-up surveys a mean of 2.08 (SD 0.31, range 1.70–2.80) years after initial application. The 22 awardees completing surveys at both time points reported improved ratings of well-being (from 4.27 [1.49] to 3.05 [1.70], *p* = 0.015, on the 7-item PWBI and from 3.45 [1.97] to 1.82 [2.17], *p* = 0.012, on the 9-item PWBI) and decreased family caregiving burden (from 37.0 [7.52] to 30.1 [8.76], *p* = 0.010). While a minimum important difference (MID) has not been established for the PWBI, scores on the 7-item PWBI improved from exceeding the established cutoff of 4 for low quality of life to below the cutoff [11]. Similarly, while no MID has been established for the BSFC-s, extrapolating from the published version (with a range of 0–30) to our modified version (with a range of 10–50) indicates that awardee ratings changed from severe to the cutoff between moderate and severe [10]. Ratings of POS did not change (24.9 [1.70] at baseline and 24.0 [2.00] at follow-up, *p* = 0.117). Taken together, results suggest that *CARES at UAB* may have provided a coping mechanism in a work environment that otherwise went unchanged, and despite improvements, caregiver burden remained in the moderate range and may warrant further intervention.

Our final codebook comprised 11 broad codes, including Personal Well-Being, Professional Impact, and Caregiving. Of these, six were further divided into 22 more detailed codes to provide a more nuanced interpretation of the data. For example, the broad code “Caregiving” was divided into four detailed codes: Partner Care, Child Care, Elder Care, and Multiple Caregiver Responsibilities. Themes and subthemes emerged from patterns in the data. From the qualitative analysis, we derived four overarching themes labeled scientific productivity, lifeline, feeling valued, and sense of community. Exemplary quotes for each theme are included in Table 2. Interview results confirmed that *CARES at UAB* helped awardees regain research productivity, and for some, served as a lifeline and accelerated progress beyond what would have been possible without funding. Recipients noted that *CARES at UAB* made them feel more noticed and valued, that the university recognized the demands on them during the height of the pandemic. Finally, awardees shared that the monthly career development seminars contributed to a sense of community among awardees, despite their belonging to different units across 4 UAB Schools.

**Table 2.** Overall themes and illustrative quotes from semi-structured interviews

Scientific Productivity
“I feel like in a lot of ways I started behind being so sick, having babies. It’s like you take one step back career-wise every time. So, CARES kind of helped me take a couple of steps forward to kind of catch up especially when you’re in this early-career stage trying to get to mid-career and you haven’t quite hit the big funding but you have a lot of stuff going on.”
“It was great to build up that momentum back again and have that study up and running and we are up to date in terms of data collection, processing of samples and everything.”
“I have folks helping every day with just small tasks like, you know, literature reviews…For me it’s maybe 2 or 3 hours of my time that I could be spending on writing.”
“I’d say we’ve been able to submit grants that we wouldn’t have otherwise been able to submit. I’d say we’ve been able to get out papers that we wouldn’t have [otherwise] published. Same thing with abstracts and national meetings”
“I’ve had the time to kind of think and breathe, to move forward with those projects. So I think it’s really just given me a lot of like mental bandwidth to not have to do [the study coordinator’s job].

Feeling Valued
‘UAB acknowledged the fact that we had just been through a lot with the pandemic, and especially as an early career researcher trying to get your career off the ground and then also as a caregiver during the pandemic. Having those things be recognized, it really made a huge difference in terms of feeling cared about, and then feeling motivated to be able to be more productive.’
“It’s some appreciation just knowing that there was institutional concern about how you are doing…It just felt good …knowing that the institution cares.”
“We all have stuff going on … outside of work that makes it hard…caregiving needs are real. We need culture change around that. It’s okay to talk about those things… I appreciate how CARES is kind of contributing to that piece.”
“I think, actually it does make you even more productive because you feel valued. You feel like people are understanding some of the challenges that you’re going through, and then you can also figure out ways to then work with those challenges.”

Sense of Community
“Hearing other people’s stories, I think, was fantastic…ways that other people have dealt with stress…I guess you know, the biggest thing is, I think, not being alone in the endeavor.”
“You are not really alone even though people’s disciplines were, you know, different. The issues of juggling and trying to maintain things when it almost seemed like things were stacked against you. It seemed to be something that was going on across the board for people. And so that did give me solace.”
“The frank discussions… that felt like a safe space… You know that other people are feeling the same way or experiencing similar challenges.”
“The seminar series that went along with CARES was probably the biggest help…hearing other people’s stories was fantastic and the ways other people have dealt with stress.”

Lifeline
‘CARES was really helpful getting me from really what would have been like a 6 month cliff, if you will. The money was running short in the lab, and we do not have any major grants coming in…CARES was helpful for paying for individuals in the lab and keeping things afloat until new grants came in.’
“I might have had to give up the pilot. I do not know if I could have done [the pilot project without CARES funding] honestly.”
”At the time [when I received the CARES funding], I didn’t have any reserves or my 2 R01s yet, and it provided security that I very much needed at the time … at that point I desperately needed it.”
“You only have certain years to get your first K. You only have certain years to get your R. So, helping us buy back some of the time, giving us some additional extra hands is very helpful.”

A thematic listing of insights and recommendations resulting from the listening sessions is included as Table 3. Briefly, tenure and promotion are a common source of confusion and stress for early-career research faculty. In some departments, information about tenure and promotion is not widely available. Strict timelines for achieving tenure and promotion are also stress-provoking. Awardees recommended that the requirements for tenure and promotion be openly communicated and that tenure tracks have more flexible timelines. In terms of balancing work and caregiving responsibilities, awardees suggested improving education about the provisions of the Family Medical Leave Act (FMLA) and how it could be utilized beyond the birth or adoption of a child. They would also like to see the creation of small grants that could be used to defray dependent and/or elder care expenses incurred when they attend out-of-town conferences. The awardees also cited the importance of and need for peer and near-peer mentorship as well as career mentorship from someone outside their primary department or school. Identifying and connecting with appropriate mentors poses many challenges. The awardees recommended the expansion of voluntary mentoring programs and the development of a database where faculty can quickly identify mentors that match their needs.

**Table 3.** Focus group themes: top concerns of round 2 and 3 awardees

Tenure and promotion	
***Insights***	***Recommendations***
Lack of transparency in the tenure and promotion processes causes concern and confusion.	Tenure and promotion expectations should be explicitly stated in materials such as employee handbooks and made widely available.
Pros and cons of tenure vs. non-tenure tracks are not clearly defined and not communicated.	Communicate the pros and cons of both tenure tracks. Offer faculty the ability to switch between tracks as their aspirations change.
Current tenure tracks (3- and 4–year tracks) are stressful and entail long hours.	Offer alternate pathways to tenure such as 5- and 6-year tenure tracks.

Family Caregiving	
***Insights***	***Recommendations***
Common misconception about FMLA is that it can only be used for the birth or adoption of a child. FMLA can be used to care aging parents, a spouse with an illness, etc.	Raise awareness about the ways FMLA and other types of leave can benefit a variety of caregivers.
Lack of flexible schedules and limited caregiving resources make it difficult to cope with unplanned situations.	Encourage departments/schools to appoint Teaching Assistants who can cover for faculty who must attend to an unplanning caregiving situation
Travel to conferences imposes a financial burden on family caregivers.	Offer local conferences and leadership positions that promote career development. Provide travel grants to offset the costs of child/elder care.

Mentoring	
**Insights**	**Recommendations**
Being matched with a mentor can be a “hit or miss” process.	Continue UAB’s voluntary Near-Peer Mentoring Program.
Finding a mentor with shared experiences/ a similar background can be challenging.	Establish a voluntary, informal mentorship program that matches mentees and mentees based on their preferences.
Locating an expert on specific topic area can be difficult.	Create a database of “ad hoc” mentors who are willing to assist early-career researchers.

## Discussion

*CARES at UAB* builds upon prior examples of targeted financial support [12–14], and the FRCS program specifically, to advance and retain early-career physician scientists at AMCs. Our results confirm many themes identified among the first *FCRS* program awardees in 2016 and in evaluations of subsequent cohorts. For example, *FRCS* funding allows awardees to delegate crucial research activities and focus on higher-level tasks such as manuscript and grant writing [15]. *FRCS* funding leads awardees to feel recognized – that institutional leadership acknowledges their work-life challenges and is trying to help them succeed [16]. *FRCS* funding creates safe spaces and community among awardees to discuss personal challenges hitherto largely hidden at work [17]. Finally, another salient theme is the biological tension between the flurry of early-career research activities and childrearing years [16], and how this particularly impacts women [18]. Takayesu and colleagues also explored the interplay between *FRCS* funding and the first months of the COVID-19 pandemic by interviewing 2017 *FRCS* awardees and analyzing narratives from 2020 *FRCS* applicants [18]. Similar to the results reported herein, they found that the pandemic exacerbated existing and significant work-life tensions and was leading to guilt and burnout, particularly among women. In terms of *COVID-19 FRCS*, a 2022 publication describes the initial program, its implementation, and early impressions from senior faculty program directors [6].

In so much, our evaluation provides new insights. To our knowledge, this evaluation is the first on awardees supported through *COVID-19 FRCS* and first to report on any *FRCS* program at an institution in the historical U.S. Deep South. Thus, our results are a glimpse into the impact of *FRCS* funding both during and immediately following the height of the COVID-19 pandemic. Our results can serve to inform and support similar philanthropic and academic responses to future events that likewise dramatically reduce time spent on research, such as natural disasters. More routinely, “extra hands” awards compliment other existing support, such as FMLA, by allowing research personnel to continue to make progress while the faculty investigator is on leave. Furthermore, *CARES at UAB* expanded *FRCS* eligibility and subsequent evaluation to non-physician research faculty in both Schools of Medicine and other health sciences facing similar caregiving demands. Unlike earlier rounds *FRCS* funding [15], *CARES at UAB* did not allow faculty to use funds to “buy out” clinical time. Funds were solely dedicated to outsourcing delegable research activities, making *CARES at UAB* generalizable to health science research faculty who do not have clinical duties. Our qualitative findings are also strengthened by the inclusion of confirmatory quantitative survey data. Additionally, we include an assessment of scientific products attributable to *CARES at UAB* funding, such as manuscripts and subsequent NIH grant acquisition. As these are the currency of productivity at AMCs, assessing these metrics may bolster leadership’s enthusiasm and support for *FRCS*-like programs. The group listening sessions to provide proactive recommendations to university leaders on how to support work-life integration are also an innovation. As compiled in Table 3, institutional leaders might be more receptive to such recommendations, beyond the benefits of “extra hands” programs, in supporting the retention and success of early-career research faculty in a post-pandemic era of academic medicine.

Finally, in the absence of available information to the contrary, we believe UAB’s legacy program to *CARES at UAB* to be novel for *FRCS* institutions. In April 2025, the UAB Heersink School of Medicine launched an intramural legacy program, *Caregivers Accelerating Research via Extra hands Support* (*HSOM CARES*). The HSOM Dean’s Office committed funds ($200,000 per year) for five years to support at least four intramural “extra hands” awards per year. Eligible recipients are HSOM early-career research faculty, physicians and non-physicians alike, that have extramural research grants covering at least 50% of their professional effort as well as significant caregiving obligations. Faculty with K-series or other extramural Career Development Awards are strongly encouraged to apply, as these awards typically provide modest support for research expenses or delegation. Based upon the interview findings reported here, we will convene monthly career development seminars for *HSOM CARES* awardees to build community and bolster morale. We will partner with other UAB Schools or Colleges should they express interest in developing analogous legacy programs.

Beyond the 5-year institutional investment in *HSOM CARES*, extramurally we are developing a structure to support the submission of NIH Administrative Supplements in response to NOT-OD-23-031 and NOT-OD-23-032 which provide supplemental funding to existing NIH K-series awardees and first-time R-series awardees, respectively, who experience a critical life event (e.g., childbirth) during their award period. Supplemental funds of up to $70,000 are for one year and are meant to support discretionary spending on “extra hands” personnel and other expenses to maintain research productivity during the critical life event. We have previously provided similar support to scholars applying for other NIH Administrative Supplements.

A number of limitations should be considered. This Special Communication describes a retention program for early-career research faculty at one AMC in the historical U.S. Deep South. The success of *CARES at UAB* may not be generalizable to all AMCs across the nation. However, unlike previous qualitative reports of the effects of the *FRCS* program on physician scientists [6,15–18] presumably from schools or colleges of medicine, this evaluation includes both physician and non-physician research faculty from four health science schools and colleges, including medicine but also nursing, public health, and health professions. Although certain challenges are unique to physicians pursuing research careers [19], the COVID-19 pandemic illuminated the tension between professional and extraprofessional obligations experienced by all research faculty with caregiving responsibilities, and therefore, our results may be more generalizable to research faculty in all of the health sciences. We expect a nationwide evaluation of all 22 *COVID-19 FRCS* sites in the near future, as alluded to in prior publications [6] and since final reports detailing outcomes of both *COVID-19 FRCS*-supported and institutionally supported awardees have been requested from all *COVID-19 FRCS* sites. Due to awardee departure from UAB and one UAB non-respondent, we were unable to interview or survey 5 and 6 awardees, respectively. Nevertheless, the interviews approached thematic saturation with the available sample size. With limited resources, the qualitative analysts were not blinded to the identity of the interviewees or transcripts. However, the analysts were of varied professional and personal backgrounds (e.g., age, race, gender, degrees of higher education). The quantitative survey improvements in well-being and decreased family caregiving demands could be attributed to the lessening of COVID-19 restrictions over time and re-opening of schools, childcare centers, adult care centers, etc. Therefore, these improvements are likely only partially attributable to *CARES at UAB*. We limited subsequent grant awards to those available in NIH Reporter to maintain consistency across our ability to track all awardees, regardless of their current institution. We acknowledge the vital importance of philanthropic, industry, and other federal research funding but are unable to systematically search for funded awards across all funding agencies as of this writing.

In conclusion, an initial evaluation of *CARES at UAB* provides another compelling case for the utilization of “extra hands” programs to support and retain early-career research faculty, physicians and non-physicians alike, with caregiving responsibilities. We hope this example of supporting non-physician research faculty from four health science schools will lead other institutions to include non-physicians in future iterations of extramural and intramural *FRCS*-like programs. Furthermore, as evidenced by awardee recommendations, university-level culture change around tenure and promotion pathways, increased flexibility for and normalization of caregiving, and expanded mentoring opportunities would be responsive to the realities of many early-career research faculty today. Such an environment is vital to cultivating harmonious work-life integration, wellness, and sustained research success, ultimately making AMCs a workplace destination of choice and retaining the talent they have devoted generations to developing.
